# Spine Metastases in Immunocompromised Mice after Intracardiac Injection of MDA-MB-231-SCP2 Breast Cancer Cells

**DOI:** 10.3390/cancers14030556

**Published:** 2022-01-22

**Authors:** Laura Brylka, Katharina Jähn-Rickert, Anke Baranowsky, Mona Neven, Michael Horn, Timur Yorgan, Harriet Wikman, Stefan Werner, Andreas Lübke, Michael Amling, Björn Busse, Klaus Pantel, Thorsten Schinke

**Affiliations:** 1 Department of Osteology and Biomechanics, University Medical Center Hamburg-Eppendorf, 20246 Hamburg, Germany; l.brylka@uke.de (L.B.); k.jaehn@uke.de (K.J.-R.); a.baranowsky@uke.de (A.B.); mo.neven@uke.de (M.N.); t.yorgan@uke.de (T.Y.); amling@uke.de (M.A.); b.busse@uke.de (B.B.); 2 Mildred Scheel Cancer Career Center, University Cancer Center Hamburg, University Medical Center Hamburg-Eppendorf, 20246 Hamburg, Germany; mi.horn@uke.de; 3 Institute of Tumor Biology, University Medical Center Hamburg-Eppendorf, 20246 Hamburg, Germany; h.wikman@uke.de (H.W.); st.werner@uke.de (S.W.); 4 Institute of Pathology, University Medical Center Hamburg-Eppendorf, 20246 Hamburg, Germany; luebke@uke.de

**Keywords:** bone remodeling, breast cancer, Kremen-2, metastasis, spine

## Abstract

**Simple Summary:**

Breast cancer cells typically metastasize to bone, where their interaction with bone remodeling cell types enhances metastatic outgrowth and osteolytic bone destruction. The respective knowledge is largely based on xenograft models, where human breast cancer cells are injected into immunocompromised mice. Importantly, however, whereas skeletal analyses in these animals are usually restricted to hindlimb bones, human skeletal metastases are far more frequent in the spine. Therefore, our study addressed the question, if breast cancer cells injected into immunocompromised mice would also metastasize to the spine, and if this process is influenced by the amount of trabecular bone. We injected an established breast cancer cell line into immunocompromised mice with or without a transgene causing severe osteoporosis. Importantly, we found more tumor cell clusters of different size in spine sections than in femora, but the presence of the transgene did not affect their spreading and metastatic outgrowth.

**Abstract:**

Breast cancer cells frequently metastasize to bone, where their interaction with bone remodeling cell types enhances osteolytic bone destruction. Importantly, however, whereas skeletal analyses of xenograft models are usually restricted to hindlimb bones, human skeletal metastases are far more frequent in the spine, where trabecular bone mass is higher compared to femur or tibia. Here, we addressed whether breast cancer cells injected into immunocompromised mice metastasize to the spine and if this process is influenced by the amount of trabecular bone. We also took advantage of mice carrying the *Col1a1-Krm2* transgene, which display severe osteoporosis. After crossing this transgene into the immunocompromised NSG background we injected MDA-MB-231-SCP2 breast cancer cells and analyzed their distribution three weeks thereafter. We identified more tumor cells and clusters of different size in spine sections than in femora, which allowed influences on bone remodeling cell types to be analyzed by comparing tumor-free to tumor-burdened areas. Unexpectedly, the *Col1a1-Krm2* transgene did not affect spreading and metastatic outgrowth of MDA-MB-231-SCP2 cells, suggesting that bone tumor interactions are more relevant at later stages of metastatic progression.

## 1. Introduction

Breast cancer cells typically metastasize to bone, where they can survive in a dormant state for several years. Once activated again, they form overt metastases, which cause local bone destruction and spreading to other organs, ultimately leading to severe insufficiencies and/or death [1,2]. The mechanisms triggering dormancy escape and metastatic outgrowth of cancer cells are not fully understood yet, but bone remodeling cell types appear to play a significant role in these processes [3,4]. In fact, the respective interactions between bone and tumor cells, commonly referred to as a vicious cycle, are key drivers of tumor cell expansion and the thereby caused osteolytic bone destruction. The strongest argument for an impact of bone turnover on metastatic outgrowth is probably given by the influence of anti-resorptive medication. More specifically, administration of bisphosphonates or blockade of the pro-osteoclastogenic cytokine RANKL were reported to reduce bone destruction in xenograft models, where human breast cancer cells are injected into immunocompromised mice and typically cause osteolytic lesions in hindlimb bones [5,6,7]. Likewise, anti-resorptive treatment of patients with skeletal metastases was reported to reduce the incidence of skeletal complications, and it was also associated with improved survival [8,9]. This explains the relevance of a large number of basic research studies, all aiming to contribute to a comprehensive understanding of the cellular interactions taking place in various phases of skeletal metastases [10].

In this regard, xenograft models have been frequently utilized to demonstrate the impact of specific molecules or cell types in an in vivo setting. More specifically, the injection of cell lines, originally obtained from different types of cancers in patients, into immunocompromised mice has been proven successful, since the majority of these cells colonize into the bone microenvironment, where they grow to form osteolytic metastases [11]. The ability to knockdown specific genes in the respective tumor cells, but also the administration of specific drugs or molecules, has allowed to identify some of the key interactions involved in the above-described vicious cycle [12,13,14,15]. It was further shown, for instance, for the commonly utilized breast cancer line MDA-MB-231, that it is possible to isolate subclones with remarkably different metastatic behavior, whose comparative analysis can generate additional knowledge regarding key molecules required for bone tropism [16]. In fact, one of the bone-seeking subclones identified, i.e., MDA-MB-231-SCP2, was used by us for the present study. Despite their value for understanding the highly relevant interactions between cancer and bone cells, xenograft experiments also have some limitations. The most obvious of these are that the molecular interactions are mediated by peptides from two different species and that the recipient mice, unlike the patients, lack a functional adaptive immune system. Another important issue is that most xenograft experiments are performed in young mice, possibly explained by the fact that older mice do not display as many skeletal lesions after tumor cell injection [17]. Finally, the vast majority of xenograft experiments are focusing on femur or tibia to determine tumor and bone quantity, as well as the extent of osteolytic bone destruction, although human breast cancer metastases are more frequently found in the spine [18]. In fact, although spine metastases have been reported to occur after injection of MDA-MB-231 cells [17,19], there is, at least to our knowledge, no published histological analysis to analyze the distribution of tumor cells in vertebral bodies.

One possible explanation for the site-specific metastatic expansion observed in patients could be that the amount of trabecular bone, and thereby the number of bone remodeling cell types, is higher in the spine than in the femur. Since the same applies for mice, we asked the question, if bone-seeking breast cancer cells would also form metastases in the spine of immunocompromised mice, and if so, whether metastatic outgrowth would be hampered by reduced trabecular bone mass. To address the latter question, we took advantage of *Col1a1-Krm2* mice, which over-express the transmembrane protein Kremen-2, a receptor for Wnt signaling inhibitors of the Dkk family, in osteoblasts [20]. On an immunocompetent genetic background, these mice develop a severe low bone mass phenotype postnatally, explained by inhibition of bone formation [21,22]. Most importantly, their trabecular bone mass in the spine was found dramatically decreased, which led us to choose this model in order to study the influence of trabecular bone mass on metastatic outgrowth.

## 2. Materials and Methods

### 2.1. Xenograft Experiments

Generation and genotyping of *Col1a1-Krm2* mice have been described previously [21]. The *Col1a1-Krm2* transgene was introduced into an immunocompromised background by backcrossing with NSG (NOD/SCID/IL2rγ^null^) mice for 10 generations. All animals used for xenografts were not only genotyped for the presence or absence of the *Col1a1-Krm2* transgene, but also for the SCID and the IL2rγ mutation. Female NSG littermates with or without the *Col1a1-Krm2* transgene (*n* = 7 of each group) were utilized for xenograft experiments. For that purpose, 10^5^ MDA-MB-231-SCP2 cells per mouse [16] were injected intracardially into the left ventricle at the age of 12 weeks. After an incubation time of 3 weeks, the respective animals were sacrificed to prepare the skeletons for further analysis, as described above for non-injected mice. The right hindlimb of each mouse was used for analyzing the bone marrow on cytospin slides, as described below. The left femur of each mouse was used for µCT scanning and histological analysis. Although two of the *Col1a1-Krm2* transgenic animals did not contain any tumor cells, neither on cytospin slides, nor histologically, unlike the other animals of the same group, we did not exclude them for the quantitative analysis of tumor cell infiltration. All experiments were approved by the animal care committees of the University Medical Center Hamburg-Eppendorf (N19/053, Org869, N17/070).

### 2.2. Skeletal Phenotyping

Dissected skeletons were fixed in 3.7% PBS-buffered formaldehyde for 18 h, before they were stored in 80% ethanol. After initial assessment by contact X-ray, the lumbar vertebral bodies L1 to L5 and the right femur were dehydrated in ascending alcohol concentrations and then embedded in Technovit 9100 for undecalcified histology according to the manufacturer’s instructions. Histological sections of 4 μm thickness from the sagittal plane were prepared, and one section each was stained by toluidine blue or von Kossa/van Gieson procedures [22]. Histomorphometry was performed according to the ASBMR guidelines using the OsteoMeasure histomorphometry system (Osteometrics Inc., Decatur, GA, USA). The left femora were used for µCT scanning using a µCT 40 desktop microCT (Scanco Medical, Wangen-Brüttisellen, Switzerland) with a voxel size of 10 µm. Reconstructed slices were analyzed using the Scanco microCT software suite. All quantitative analyses were performed in a blinded fashion. For TRAP activity staining, one sections per sample was preincubated in 10 mM sodium tartrate dissolved in 40 mM acetate buffer (pH 5). Sections were then stained with 0.1 mg/mL naphtol AS-MX phosphate (Sigma Biochemicals, N-5000, Darmstadt, Germany) in the same buffer, including 0.6 mg/mL Fast Red Violet LB salt (Sigma Biochemicals, F-3881, Darmstadt, Germany).

### 2.3. Tumor Cell Detection

For tumor cell quantification on cytospin slides, bone marrow of the right femur and tibia was isolated by centrifugation. After lysis of erythrocytes, the remaining cells were counted and centrifuged onto glass slides. Slides were stained for human pan-cytokeratin (AE1/AE3 eFlour-570, Invitrogen 41-9003-82) and CD45 (CD45-APC, BD Pharmingen 5598641:150, 30-F11, BD) to identify tumor cells with immunofluorescence. For the detection of tumor cells on histological sections, antigen retrieval was performed with Tris Buffer pH10. Sections were incubated with anti-human cytokeratin antibody AE1/AE3 (Agilent, M351501, Santa Clara, CA, USA) and a detection kit was used to amplify and visualize the staining (Vector, MP-7802-15, Burlingame, CA, USA). For each mouse, we also stained one section of the left femur with toluidine blue, where tumor areas were quantified using the OsteoMeasure histomorphometry system (Osteometrics Inc., Decatur, GA, USA). Pseudocolor images were created by manually tracing bone, bone marrow and tumor cells according to their morphology. The same method was applied for quantification of the tumor cell area in individual vertebral bodies (L1 to L5). Additionally, three non-serial sections of the spine were used for quantification of the tumor areas by ImageJ.

### 2.4. Osteocyte Staining

Ploton silver staining for osteocyte visualization was performed on sections of vertebral bone tissue embedded in Technovit 9100. Sections were decalcified in 20% ethylenediaminetetraacetic solution for 30 min, incubated with a 50% silver nitrate solution for 55 min before counterstaining in a 5% sodium thiosulfate solution for 10 min. The number of empty lacunae over the total lacuna number was determined in cortical and trabecular bone. A total number above 100 lacunae per region of interest was analyzed using the Osteomeasure system. For each mouse, we analyzed one section of five vertebral bodies (L1–L5).

### 2.5. Serum Analysis

For the determination of bone turnover biomarkers, serum of the sacrificed mice was collected immediately *post mortem*. Quantification was performed using antibody-based detection kits according to the manufacturer’s instructions (CTX-1: AC-06F1, Immunodiagnostic Systems, UK; PINP: SEA957Mu, Cloud Clone Corp., Katy, TX, USA).

### 2.6. Data Analysis

All data are presented as mean ± standard deviations. Statistical analysis was performed using unpaired, two-tailed Student’s *t*-test. For the comparison of more than two groups, one-way ANOVA with Tukey correction was applied. *p*-values below 0.05 were considered statistically significant.

## 3. Results

### 3.1. The Col1a1-Krm2 Transgene Does Not Affect Metastatic Outgrowth of Bone-Seeking Breast Cancer Cells in Femora of NSG Mice

**Figure 1:**
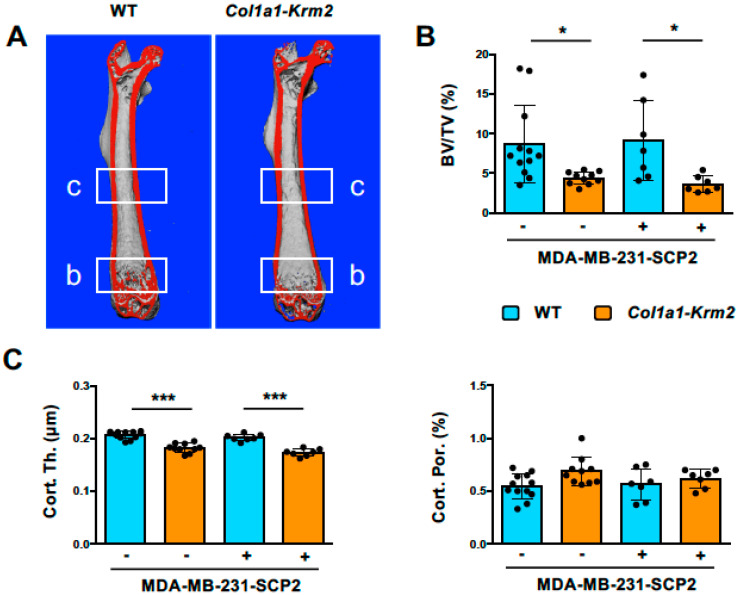
The *Col1a1-Krm2* transgene confers low trabecular and cortical bone mass to NSG mice. (**A**) Representative µCT scans of femoral bones from NSG mice (WT) and *Col1a1-Krm2*-transgenic NSG mice (*Col1a1-Krm2*) three weeks after injection of MDA-MB-231-SCP2 cells. The indicated regions were analyzed for quantification of the trabecular or cortical bone mass. (**B**) Quantification of the trabecular bone volume per tissue volume (BV/TV) in the two groups of mice with (+) or without (−) injected cancer cells. (**C**) Quantification of cortical thickness (Cort. Th.) and porosity (Cort. Por.) in the same groups of mice. Data represent mean ± standard deviation (*n* ≥ 7). Statistical significance was determined by one-way ANOVA; * *p* < 0.05, *** *p* < 0.0005.

The *Col1a1-Krm2* transgene was introduced into an immunocompromised background by backcrossing with NSG (NOD/SCID/IL2rγ^null^) mice for 10 generations. Female NSG littermates with or without the *Col1a1-Krm2* transgene were utilized for xenograft experiments. For that purpose, MDA-MB-231-SCP2 cells, representing a bone-seeking subclone of an established breast cancer cell line [16], were injected intracardially into 12-week-old mice and the respective animals were analyzed 3 weeks thereafter. This relatively short standing time was chosen, since we intended to focus on early metastatic stages without excessive osteolytic lesions. We first performed µCT scanning of femoral bones to analyze trabecular and cortical bone parameters in injected and non-injected animals of both genotypes (Figure 1A). We observed the expected reduction in the trabecular bone volume in *Col1a1-Krm2*-transgenic NSG mice compared to NSG controls, but there was no further impact caused by the injection of tumor cells (Figure 1B). The same was the case for the femur cortical thickness, whereas cortical porosity was not influenced, neither by genotype nor by injection of tumor cells (Figure 1C).

**Figure 2:**
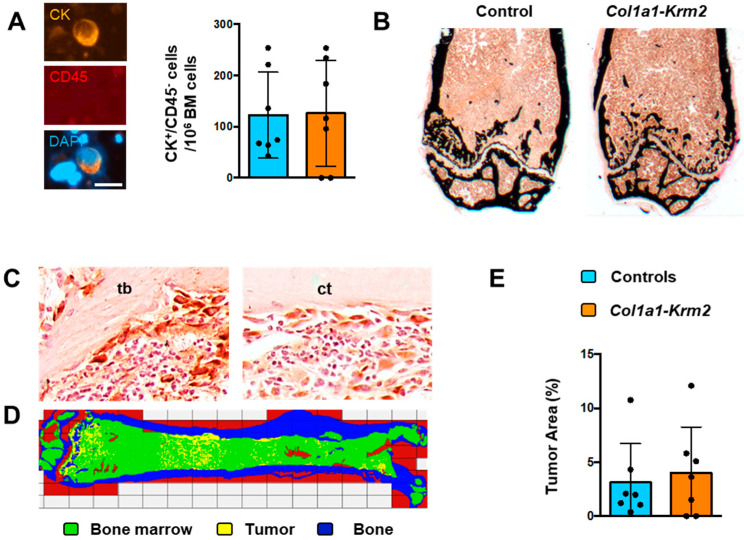
The *Col1a1-Krm2* transgene does not affect metastatic outgrowth in femora of NSG mice. (**A**) Staining (left) and quantification (right) of human breast cancer cells (positive for cytokeratin, CK; negative for CD45) on cytospin slides from bone marrow of NSG mice (WT) and *Col1a1-Krm2*-transgenic NSG mice (*Col1a1-Krm2*) three weeks after injection of MDA-MB-231-SCP2 cells. (**B**) Representative von Kossa staining of undecalcified femur sections from the same mice. Mineralized bone is stained in black. (**C**) Representative immunohistochemistry with a cytokeratin-specific antibody for detection of human breast cancer cells in trabecular (tb) and cortical (ct) bone regions in femur sections of an injected NSG mouse. (**D**) Tumor cell distribution in a representative femur of an injected NSG mouse. Areas representing bone, unaffected bone marrow or infiltrated tumor cells are indicated by pseudocolours. (**E**) Quantification of the tumor area in femur sections of all experimental mice. Data represent mean ± standard deviation (*n* = 7). Statistical significance was determined by Student’s *t*-test.

For tumor cell quantification, bone marrow of the right femur and tibia was isolated by centrifugation and analyzed on cytospin slides. Here, we did not observe a significant difference in the number of CK^+^/CD45^−^ cancer cells between NSG mice and *Col1a1-Krm2*-transgenic NSG mice (Figure 2A). We further analyzed the left femur by undecalcified histology (Figure 2B). Although we did not yet observe any signs of severe osteolysis in both groups of mice, we identified tumor cell clusters in mice of both genotypes, which was confirmed by immunohistochemistry with cytokeratin-specific antibodies (Figure 2C). Of note, tumor-infiltrated areas were not only found in the metaphyseal regions containing trabecular bone, but also in diaphyseal regions where only a cortical shell is present (Figure 2D, Supplementary Figure S1). It is also critical to state that we did not identify tumor cells in two of the *Col1a1-Krm2*-transgenic NSG mice. Most importantly, however, quantification of the tumor cell area did not identify a significant difference between NSG mice and *Col1a1-Krm2*-transgenic NSG mice, even if all mice were included in the statistical analysis (Figure 2E).

### 3.2. MDA-MB-231-SCP2 Cells Form Various Metastases in the Lumbar Spine Independent of Trabecular Bone Mass

**Figure 3:**
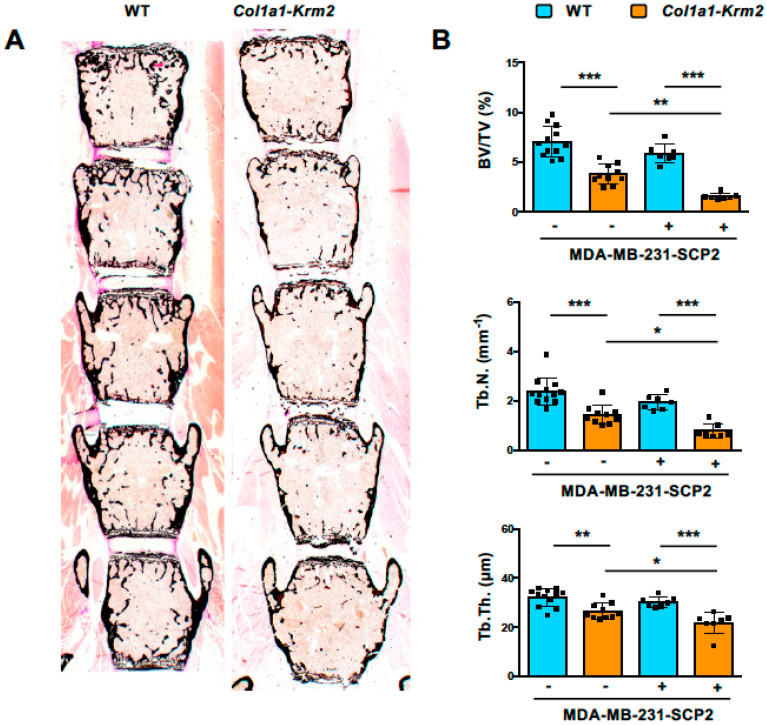
*Col1a1-Krm2*-transgenic NSG mice display strongly reduced trabecular bone mass in the spine. (**A**) Representative von Kossa staining of undecalcified lumbar spine sections from NSG mice (WT) and *Col1a1-Krm2*-transgenic NSG mice (*Col1a1-Krm2*) three weeks after injection of MDA-MB-231-SCP2 cells. Mineralized bone is stained in black. (**B**) Histomorphometric quantification of the trabecular bone volume per tissue volume (BV/TV), trabecular number (Tb.N.) and trabecular thickness (Tb.Th.) in the two groups of mice with (+) or without (−) injected cancer cells. Data represent mean ± standard deviation (*n* ≥ 7). Statistical significance was determined by one-way ANOVA; * *p* < 0.05, ** *p* < 0.005, *** *p* < 0.0005.

The major purpose of our investigation was to analyze to what extent the injected MDA-MB-231-SCP2 cells would form metastases in the spine, where trabecular bone mass, when referring to the entire skeletal element, is much higher than in hindlimb bones. By analyzing undecalcified sections of the lumbar vertebral bodies L1 to L5, we observed that the trabecular bone mass was strongly reduced by the presence of the *Col1a1-Krm2* transgene (Figure 3A), similar to what we have previously reported for this model on an immunocompetent genetic background [21,22]. Histomorphometric quantification confirmed this observation and identified significant genotype differences in all three trabecular bone parameters, i.e., trabecular bone volume per tissue volume, trabecular number and trabecular thickness (Figure 3B). Moreover, these parameters were further reduced in *Col1a1-Krm2*-transgenic NSG mice by tumor cell injection.

**Figure 4:**
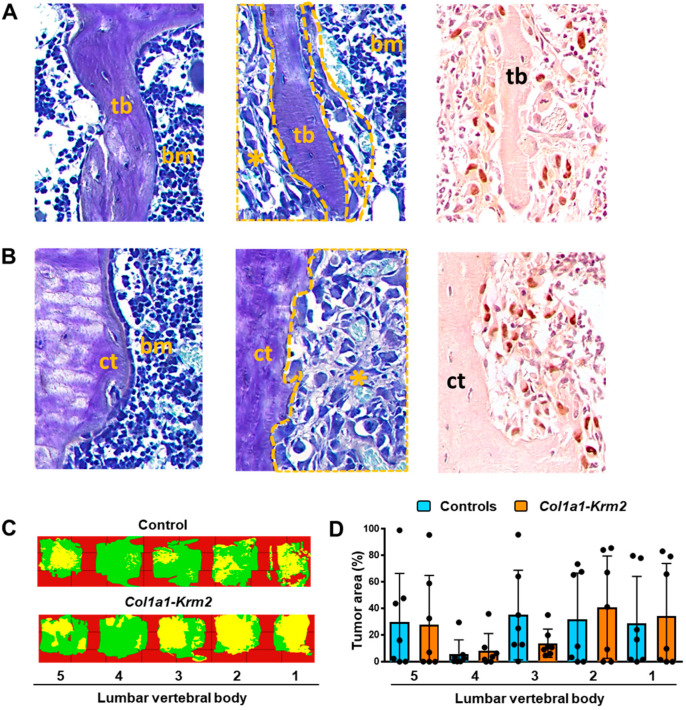
The *Col1a1-Krm2* transgene does not affect metastatic outgrowth in the lumbar spine of NSG mice. (**A**) Representative toluidine blue staining of trabecular bone (tb) areas in undecalcified lumbar spine sections from an injected NSG mouse. The panels show tumor-free bone areas surrounded by bone marrow (bm) (left), tumor-burdened areas (*) (middle) and immunohistochemistry with a cytokeratin-specific antibody (right). (**B**) Representative toluidine blue staining of cortical bone (ct) areas in the same sections. The panels are organized as described in (**A**). (**C**) Tumor cell distribution in representative lumbar spines of an injected NSG and *Col1a1-Krm2*-transgenic NSG mouse. Areas representing unaffected bone marrow (green) or infiltrated tumor cells (yellow) are indicated by pseudocolours. (**D**) Quantification of the tumor area in individual lumbar vertebral bodies (L1 to L5) of all experimental mice. Data represent mean ± standard deviation (*n* = 7). Statistical significance was determined by Student’s *t*-test.

Most importantly, however, we identified numerous metastases in lumbar vertebral bodies from NSG mice of both genotypes. These were visible in trabecular (Figure 4A) and cortical bone areas (Figure 4B), either by toluidine blue staining or by immunohistochemistry with cytokeratin-specific antibodies. Overall, metastases of varying sizes were randomly distributed, and were identified, with few exceptions, in all of the analyzed lumbar vertebral bodies (Figure 4C, Supplementary Figure S2). Although our quantitative analysis revealed a large heterogeneity in the volume occupied by tumor cells, the quantification of the tumor area did not reveal significant differences between NSG mice and *Col1a1-Krm2*-transgenic NSG mice, also not at the level of individual vertebral bodies (Figure 4D). Likewise, there were no significant differences observed between the two genotypes, when we compared three non-serial sections of the lumbar spine (Table S1). To our best knowledge, these data provide the first histological evidence that xenografts of breast cancer cells also cause metastases in the spine.

### 3.3. MDA-MB-231-SCP2 Metastases Affect Osteoclastogenesis and the Osteocyte Network

**Figure 5:**
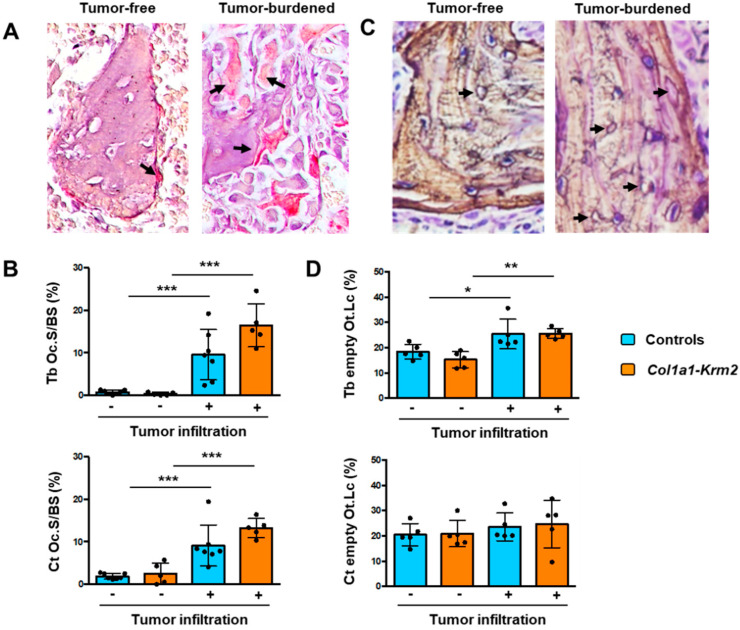
MDA-MB-231-SCP2 metastases affect osteoclastogenesis and the osteocyte network. (**A**) Representative TRAP activity staining of osteoclasts (arrows) on spine sections from an NSG mouse three weeks after injection of MDA-MB-231-SCP2 cells. The left panel represents a trabecular bone area without tumor cell infiltration, whereas the right panel shows trabecular bone in a tumor-burdened area. Arrows indicate TRAP-positive osteoclasts. (**B**) Quantification of the osteoclast surface per bone surface (Oc.S/BS) at trabecular (Tb) or cortical (Ct) bone in tumor-free (−) and tumor-burdened (+) areas from all experimental mice. (**C**) Representative silver staining of undecalcified spine sections from an NSG mouse three weeks after injection of MDA-MB-231-SCP2 cells. The left panel represents a trabecular bone area without tumor cell infiltration, whereas the right panel shows trabecular bone in a tumor-burdened area. Arrows indicate empty osteocyte lacunae. (**D**) Quantification of the percentage of empty osteocyte lacunae (Ot.Lc) within trabecular (Tb) or cortical (Ct) bone in tumor-free (−) and tumor-burdened (+) areas from all experimental mice. Data represent mean ± standard deviation (*n* = 5). Statistical significance was determined by one-way ANOVA; * *p* < 0.05, **p < 0.005, *** *p* < 0.0005.

One major advantage of analyzing spine instead of femur sections is that many different sites can be evaluated for each animal and that areas with or without tumor cells can be histomorphometrically compared. In fact, based on toluidine blue staining it was obvious that the number of osteoclasts was strongly increased at trabecular bone surfaces present in metastatic areas. To confirm this observation, we applied TRAP activity staining and quantified osteoclasts in areas with or without tumor metastases (Figure 5A). Here, we detected a more than 4-fold increase in the osteoclast surface per trabecular or cortical bone surface in metastatic areas, and again this tumor cell influence was not affected by the *Col1a1-Krm2* transgene (Figure 5B). We also noticed that the number of empty osteocyte lacunae was increased in tumor-burdened trabecular bone areas in NSG mice of both genotypes (Figure 5C). This was also confirmed by quantitative analysis, but this tumor-mediated influence on the osteocyte network was only observed in trabecular, not in cortical bone (Figure 5D).

**Figure 6:**
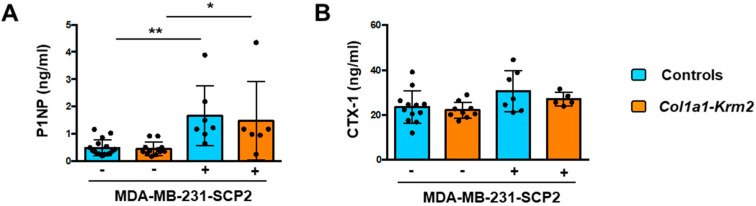
MDA-MB-231-SCP2 metastases cause a systemic activation of bone formation. (**A**) Serum concentrations of PINP in NSG mice (WT) and *Col1a1-Krm2*-transgenic NSG mice (*Col1a1-Krm2*) three weeks after injection of MDA-MB-231-SCP2 cells. (**B**) Serum concentrations of CTX-1 in the same mice. Data represent mean ± standard deviation (*n* ≥ 5). Statistical significance was determined by one-way ANOVA; * *p* < 0.05, ** *p* < 0.005.

Since matrix-embedded osteocytes are known as key regulators of bone remodeling, we finally measured the serum concentrations of two established biomarkers to monitor bone formation (PINP) or bone resorption (CTX-1). With respect to PINP, representing the N-terminal propeptide of type I procollagen, we observed significantly higher levels in mice that received tumor cell injections, indicative of excessive osteoblast activity (Figure 6A). In contrast, serum concentrations of CTX-1, representing the C-telopeptide of type I collagen, were not significantly different between the two groups, thereby revealing that the local activation of osteoclastogenesis by tumor cells did not translate into systemically increased bone resorption rates (Figure 6B). Taken together, our data demonstrate that the injected breast cancer cells did not only form metastases in vertebral bodies, but also mediated strong influences on all bone remodeling cell types, consistent with the concept of the vicious cycle that eventually results in osteolytic bone destruction.

## 4. Discussion

Bone remodeling is a life-long physiologically relevant process that is required for long-term skeletal integrity [23]. The two cell types mediating bone remodeling, i.e., bone-forming osteoblasts and bone-resorbing osteoclasts, are fundamentally different in terms of progenitor cells and mode of action, which also explains that there are regulated by different sets of molecules [24,25]. Some of these molecules are produced by osteocytes, representing the most abundant cell population in the skeleton, which are derived from osteoblasts and form a cellular network within the mineralized bone matrix [26]. Since skeletal metastases are frequently observed in the three most common types of carcinomas (breast, prostate, lung), it is obvious that a comprehensive understanding of the cellular interactions between cancer and bone remodeling cells will not only be informative in terms of basic science, but also from a therapeutic perspective.

In addition to the adverse effects of tumor cells on the skeleton, there is accumulating evidence for a detrimental molecular crosstalk between bone and tumor cells, which is often depicted as a vicious cycle [4]. Many of the respective findings on this cellular interaction were obtained by xenograft experiments. The vast majority of the respective studies are focused on later stages of metastatic outgrowth, where activated osteoclastogenesis and osteolytic lesioning is studied in hindlimb bones, i.e., femur or tibia. Importantly, however, human breast cancer metastases are more frequently found at other locations, including the spine, and the transferability of findings from xenograft experiments to human pathology is further hampered by the use of rather young animals in such studies [17]. We therefore aimed at modifying this analysis by using adult mice, focusing on earlier stages of metastatic progression and, most importantly, performing histomorphometric quantification not only in sections of the femur, but also of the spine. Finally, to address the question if metastatic outgrowth of breast cancer cells depends on the amount of trabecular bone, we took advantage of *Col1a1-Krm2* mice, since these mice develop severe postnatal osteoporosis [21,22].

Our results clearly showed that the injected MDA-MB-231-SCP2 cells led to metastatic outgrowth not only in femoral bones, but also in vertebral bodies of NSG mice, and that this process was apparently not affected by the presence of the *Col1a1-Krm2* transgene. Importantly, however, the heterogeneity of tumor cell distribution that we observed was far too high to reach a sufficient statistical power. More specifically, a post hoc analysis using the G*power software revealed that a number of 150 animal per group would have been required draw a definite conclusion, i.e., that spreading and metastatic outgrowth of MDA-MB-231-SCP2 cells is not significantly influenced by trabecular bone mass. Regardless of this limitation, we also took advantage of the vertebral body sections to compare bone areas with or without tumor cell infiltration. We hereby observed remarkable differences in the number of osteoclasts, again independent of the *Col1a1-Krm2* genotype, which essentially confirms previous data showing that breast cancer cells activate osteoclastogenesis to ultimately cause osteolytic lesions [8]. Moreover, we observed a less pronounced, yet significant difference between tumor-affected and unaffected areas in terms of empty osteocyte lacunae. These were increased in trabecular bone structures of tumor-burdened areas in both experimental groups, suggesting an impact of metastases on the osteocyte network, which provides the basis for further investigations. In this context, osteocyte apoptosis may play a significant role in the activation of osteoclastogenesis [27,28]. Moreover, since osteocytes are major orchestrators of bone remodeling, for instance by producing the anti-osteoanabolic molecule Sclerostin, their affection by MDA-MB-231-SCP2 metastases may also explain the systemically increased bone formation in the injected mice [29].

On the other hand, since there was no difference between NSG mice and *Col1a1-Krm2*-transgenic NSG mice in terms of tumor cell colonization and early metastatic outgrowth, it appears that interactions between bone and cancer cells are more relevant at later stages of metastases. The fact that tumor colonies have to reach a particular size before a localized influence on bone remodeling cells is detectable, has previously been reported in a detailed histological analysis of hindlegs at different time points after intracardiac injection of MDA-MB-231 cells [30]. In this context it is also noteworthy that a recent retrospective cohort study of breast cancer patients has demonstrated, in line with our findings, that women with untreated precancer osteoporosis did not have an increased risk for bone metastasis [31].

However, we certainly cannot draw generalized conclusions with respect to the vicious cycle, since we only analyzed one breast cancer cell line, which was chosen due to its established bone tropism [16]. Therefore, since our established protocol for immunodetection of human breast cancer cells on undecalcified bone sections of recipient mice also allows the detection of disseminated tumor cells, it will be important to perform a similar experiment with less aggressive breast cancer cell lines, such as the SCP6 subclone of MDA-MB-231, which does not cause severe osteolytic lesions [16]. Moreover, although this requires time-consuming backcrosses into the NSG genetic background, it might be worthwhile to perform such xenograft experiments with mice carrying other genetic modifications. In fact, it would be interesting to monitor metastatic outgrowth of different cancer cell lines in NSG mice with osteoblast or osteoclast activation.

Despite the many limitations of our study, our results clearly demonstrate that the spine is an excellent site to evaluate metastatic outgrowth of human cancer cell lines in xenograft experiments and to uncover their molecular interactions with bone remodeling cell types. In fact, since several metastases of varying size can be found in different vertebral bodies of individual animals, not only the comparison of affected and non-affected areas is highly informative. Moreover, a comparison of small and large metastases in terms of their potential crosstalk with each other and the surrounding microenvironment should be achievable. This could be done at different stages after tumor cell injection, also in older mice, and, most importantly, by comparing different cell lines.

## 5. Conclusions

There are several novel and important aspects of our study. First, to our best knowledge, we provide the first histological evidence that xenografts of breast cancer cells also cause metastases in the spine, which mimics the situation in cancer patients. Second, we demonstrate that spreading and metastatic outgrowth of an established bone-seeking breast cancer cell line is not influenced by trabecular bone mass, i.e., by the number of bone remodeling cell types. Third, we identified specific influences of breast cancer cells on osteoclastogenesis and the osteocyte network by comparing tumor-free to tumor-burdened areas. Finally, our combined analyses identify the lumbar spine as an ideal readout site to study bone tumor cell interactions in xenograft experiments.

## Data Availability

The data sets used and analyzed in the current study are available.

## Supplementary Materials

The following are available online at https://www.mdpi.com/article/10.3390/cancers14030556/s1, Figure S1: Summary views of histological femora sections of all injected NSG mice of the indicated genotypes with histologically detectable tumor cells. Figure S2: Summary views of histological lumbar spine sections of all injected NSG mice of the indicated genotypes with histologically detectable tumor cells. Table S1: Quantification of the tumor area in three non-serial sections of the lumbar spine for all experimental mice.
