# Supplementary material for: Spine Metastases in Immunocompromised Mice after Intracardiac Injection of MDA-MB-231-SCP2 Breast Cancer Cells
Source: Cancers (Basel). 2022 Jan 22;14(3):556. doi: 10.3390/cancers14030556 (PMC8833437; doi:10.3390/cancers14030556)
Supplement: Supplementary file 1 [file cancers-14-00556-s001.zip › cancers-1536683-supplementary.pdf]

Supplementary Materials

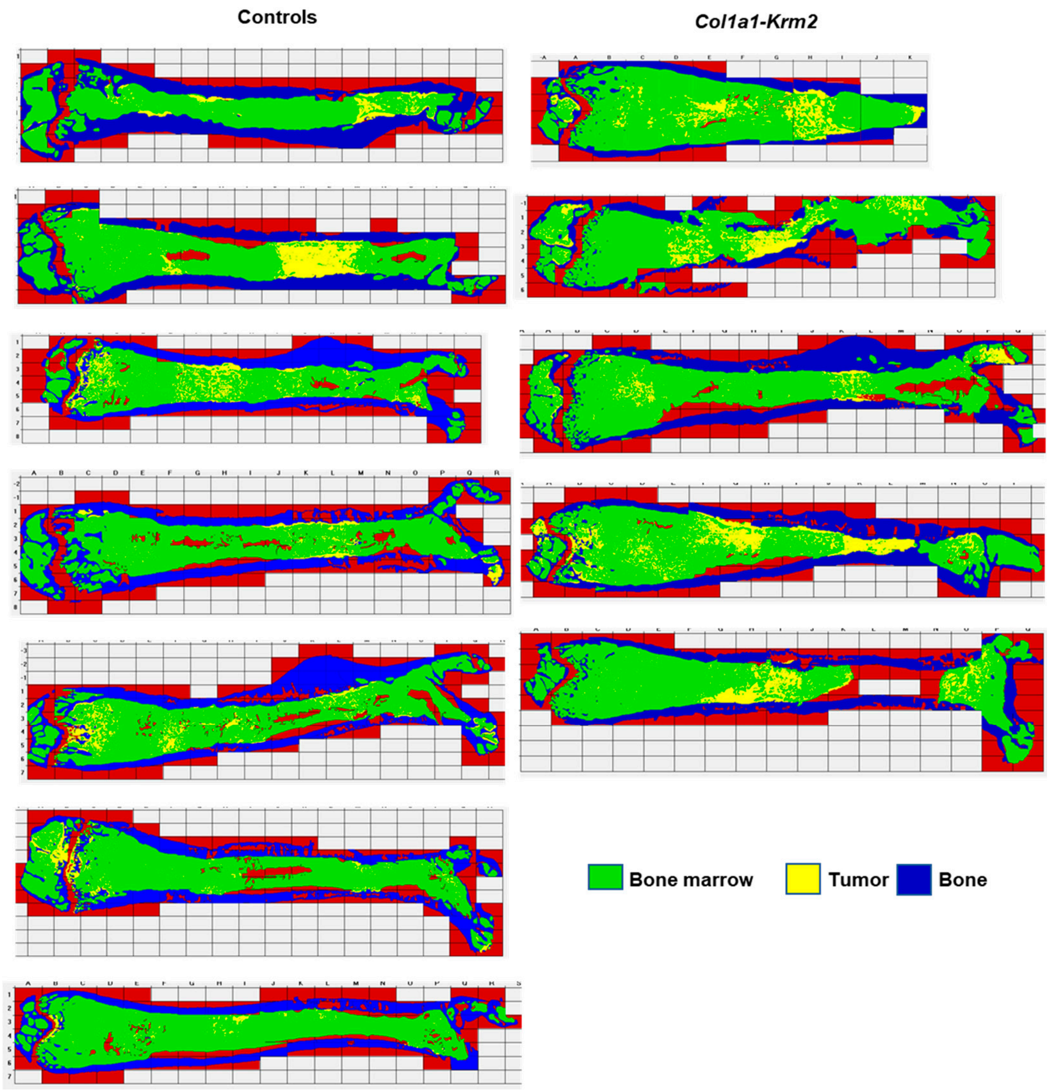

Figure S1. Summary views of histological femora sections of all injected NSG mice of the indicated genotypes with histologically detectabele tumor cells. The sections were analyzed using the Osteomeasure system, and areas representing bone, unaffected bone marrow or infiltrated tumor cells are indicated by pseudocolours.

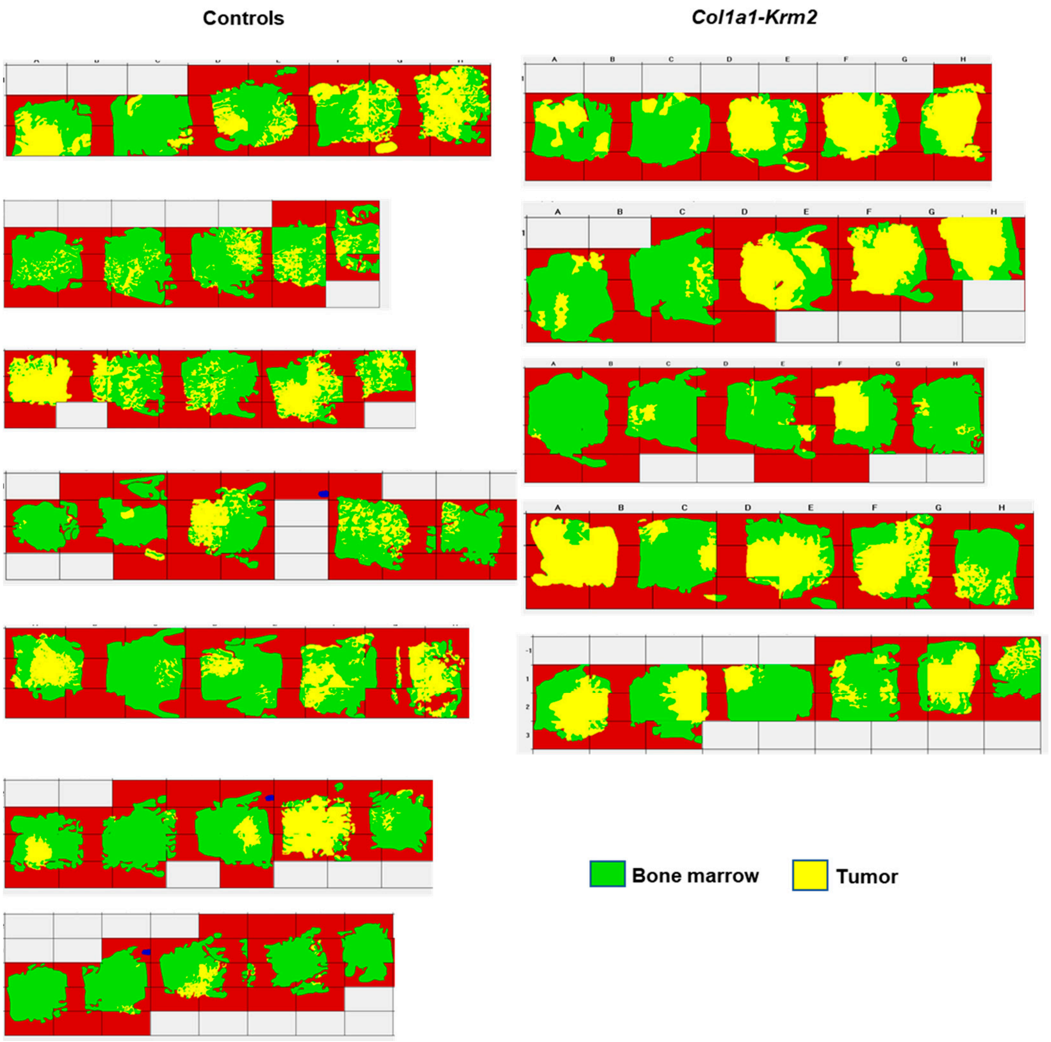

Figure S2. Summary views of histological lumbar spine sections of all injected NSG mice of the indicated genotypes with histologically detectabele tumor cells. The sections were analyzed using the Osteomeasure system, and areas representing bone marrow or infiltrated tumor cells are indicated by pseudocolours.

	control L1	*Col1a1-Krm2* L1	control L2	*Col1a1-Krm2* L2	control L3	*Col1a1-Krm2* L3	control L4	*Col1a1-Krm2* L4	control L5	*Col1a1-Krm2* L5
	91,122	69,882	83,684	60,031	30,219	48,876	3,037	6,710	41,723	25,239
**#1**	85,305	72,177	56,544	74,671	45,758	53,876	4,879	12,150	44,915	23,604
	89,430	71,707	57,766	63,216	58,843	44,876	3,838	12,488	53,253	20,064
	50,233	73,880	21,933	76,203	68,140	79,405	28,750	11,535	23,611	5,361
**#2**	63,562	71,969	43,004	65,573	40,067	56,800	39,065	14,538	35,499	15,760
	33,669	72,535	40,747	68,148	36,230	73,604	24,338	11,967	23,428	7,787
	62,164	8,205	63,023	36,179	37,196	23,946	63,341	8,266	95,905	0,558
**#3**	71,990	1,866	69,330	39,678	32,296	15,251	61,133	10,164	94,579	0,000
	30,672	10,084	69,119	39,808	16,728	17,748	73,472	4,799	97,323	0,000
	15,852	40,540	50,538	60,523	66,230	58,030	13,196	12,554	5,962	83,662
**#4**	15,825	34,647	37,552	65,295	60,470	60,199	14,895	11,674	5,506	90,838
	14,185	35,460	34,484	65,459	54,563	54,871	10,631	14,255	10,154	84,087
	83,559	65,067	38,078	35,453	21,697	16,639	4,181	33,592	42,312	49,876
**#5**	86,215	56,525	51,346	26,606	21,649	14,448	7,862	33,380	46,940	48,699
	86,190	66,151	53,340	47,102	16,131	15,725	5,447	37,815	32,569	52,521
	20,199	0,000	88,839	0,000	9,656	0,000	2,737	0,000	18,969	0,000
**#6**	25,399	0,000	87,430	0,000	14,119	0,000	9,792	0,000	23,859	0,000
	26,574	0,000	88,853	0,000	12,407	0,000	15,071	0,000	24,451	0,000
	0,000	0,000	5,711	0,000	28,079	0,000	5,274	0,000	1,500	0,000
**#7**	5,259	0,000	15,248	0,000	26,010	0,000	3,138	0,000	0,642	0,000
	0,449	0,000	5,748	0,000	25,485	0,000	2,589	0,000	0,000	0,000
**Mean ± SD**	45,612 ± 32,298	35,747 ± 31,150	50,587 ± 25,033	39,236 ± 27,998	34,380 ± 17,857	30,205 ± 26,683	18,889 ± 21,426	11,233 ± 11,022	34,433 ± 29,624	24,193 ± 30,537

Supplemental Table 1. Quantification of the tumor area in three non-serial sections of the lumbar spine for all experimental mice (n=7). Values represent the tumor area in individual vertebral bodies (L1 to L5) as a percentage of the bone marrow area. No significant genotype-dependent differences were observed, as determined by Student´s *t*-test.
